# [Tris(3,5-diphenyl­pyrazol­yl)hydro­borato]nickel(II) bromide

**DOI:** 10.1107/S1600536809021606

**Published:** 2009-06-13

**Authors:** David J. Harding, Phimphaka Harding, Harry Adams

**Affiliations:** a Molecular Technology Research Unit, Department of Chemistry, Walailak University, Thasala, Nakhon Si Thammarat 80161, Thailand; b Department of Chemistry, Faculty of Science, University of Sheffield, Brook Hill, Sheffield S3 7HF, England

## Abstract

In the title tris­(pyrazol­yl)borate (Tp^Ph2^) complex, [NiBr(C_45_H_34_BN_6_)], the Ni, Br and B atoms lie on a crystallographic threefold axis and a distorted NiN_3_Br tetra­hedral geometry arises for the metal ion. In the crystal, C—H⋯(C=C) and C—H⋯π inter­actions help to establish the polar crystal packing.

## Related literature

For other Tp^*R*^Ni*X* (*X* = Cl, Br) complexes, see: Desrochers *et al.* (2003 ▶, 2006 ▶); Kunrath *et al.* (2003 ▶); Uehara *et al.* (2002 ▶); Guo *et al.* (1998 ▶); Harding *et al.* (2007 ▶). For ionic radius data, see: Shannon (1976 ▶).
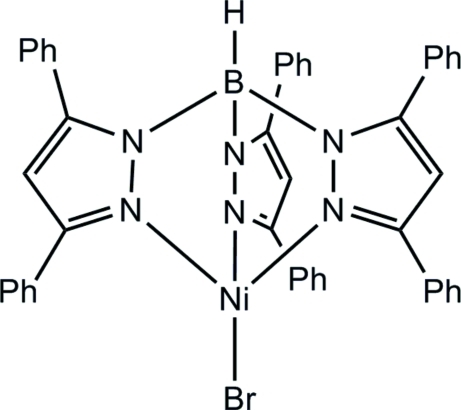


## Experimental

### 

#### Crystal data

[NiBr(C_45_H_34_BN_6_)]
*M* *_r_* = 808.21
Trigonal, 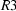

*a* = 12.8227 (8) Å
*c* = 19.327 (3) Å
*V* = 2752.0 (5) Å^3^
*Z* = 3
Mo *K*α radiation
μ = 1.66 mm^−1^
*T* = 150 K
0.24 × 0.24 × 0.21 mm

#### Data collection

Bruker SMART CCD diffractometer
Absorption correction: multi-scan (*SADABS*; Bruker, 1997 ▶) *T*_min_ = 0.691, *T*_max_ = 0.722
5609 measured reflections
2075 independent reflections
1943 reflections with *I* > 2σ(*I*)
*R*_int_ = 0.037

#### Refinement

*R*[*F* ^2^ > 2σ(*F* ^2^)] = 0.028
*wR*(*F* ^2^) = 0.063
*S* = 1.06
2075 reflections
163 parameters
1 restraint
H-atom parameters constrained
Δρ_max_ = 0.27 e Å^−3^
Δρ_min_ = −0.29 e Å^−3^
Absolute structure: Flack (1983 ▶), 670 Friedel pairs
Flack parameter: 0.020 (8)

### 

Data collection: *SMART* (Bruker, 1997 ▶); cell refinement: *SAINT* (Bruker, 1997 ▶); data reduction: *SAINT*; program(s) used to solve structure: *SHELXS97* (Sheldrick, 2008 ▶); program(s) used to refine structure: *SHELXL97* (Sheldrick, 2008 ▶); molecular graphics: *SHELXTL* (Sheldrick, 2008 ▶); software used to prepare material for publication: *SHELXTL*.

## Supplementary Material

Crystal structure: contains datablocks I, global. DOI: 10.1107/S1600536809021606/hb2976sup1.cif

Structure factors: contains datablocks I. DOI: 10.1107/S1600536809021606/hb2976Isup2.hkl

Additional supplementary materials:  crystallographic information; 3D view; checkCIF report

## Figures and Tables

**Table:** 

Ni1—Br1	2.3523 (6)
Ni1—N1	2.041 (2)

**Table:** 

N1—Ni1—N1^i^	93.11 (8)
N1—Ni1—Br1	123.04 (6)

Symmetry code: (i) 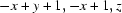.

**Table 2:** Hydrogen-bond geometry (Å, °)

*D*—H⋯*A*	*D*—H	H⋯*A*	*D*⋯*A*	*D*—H⋯*A*
C5—H5⋯*Cg*1^ii^	0.95	2.73	3.589 (3)	151

Symmetry code: (ii) 
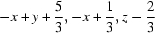
.

**Fig. 1.**
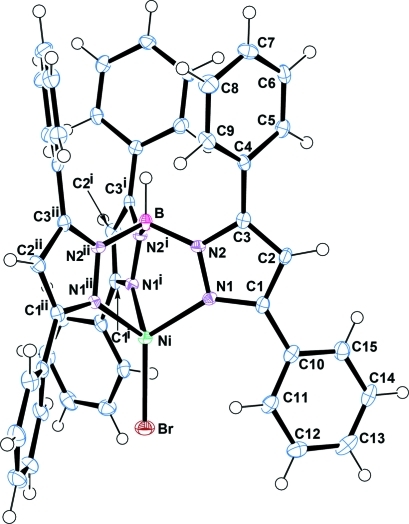
The molecular structure of (I) with displacement ellipsoids drawn at the 50% probability level. [Symmetry codes:(i) -x + y+1, -x + 1, z; (ii) -y + 1, x-y, z].

**Fig. 2.**
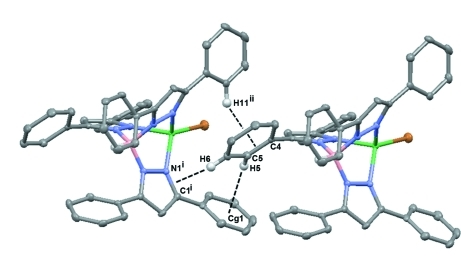
The molecular packing in (I) showing the three C—H···π interactions. Only selected atoms are labelled or shown for clairty [Symmetry codes:(i) 4/3 - x + y, 2/3 - x, -1/3 + z; (ii) 4/3 - y, -1/3 + x-y, -1/3 + z].

## supplementary crystallographic information

### Comment

Tris(pyrazolyl)borates are versatile and popular ligands in coordination chemistry with many complexes now known. Despite the C_3_ symmetry present in many tris(pyrazolyl)borate ligands few tetrahedral complexes crystallize in space groups containing a C_3_ axis (Desrochers *et al.*, 2003, 2006; Kunrath *et al.*, 2003; Uehara *et al.*, 2002). Rare examples which do contain a C_3_ axis include [Tp^Ph2^NiCl] and [Tp^Ph2^Ni(OAc)] despite the later being formally five-coordinate (Guo *et al.*, 1998; Harding *et al.*, 2007). In the following paper we report a further example namely, the title compound, [Tp^Ph2^NiBr], (I).

The reaction of NiBr_2_.2H_2_O with KTp^Ph2^ readily affords the title complex as a red-purple solid in moderate yield. Crystals were grown by allowing hexanes to diffuse into a concentrated solution of the complex in CH_2_Cl_2_. The compound crystallizes in the trigonal *R3* space group. The structure is shown in Figure 1 while important bond lengths and angles are given in the supporting tables. The geometry around the nickel centre is best described as distorted tetrahedral {N1—Ni—N1^i^ = 93.11 (8), N1—Ni—Br1 = 123.04 (6)}. The Ni—N bond lengths are very slightly longer by *ca*. 0.01 Å than those found in [Tp^Ph2^NiCl] (Guo *et al.*, 1998). A similar difference is observed in the structures of [Tp*NiCl] and [Tp*NiBr] (Desrochers *et al.*, 2003, 2006). The Ni—Br distance is 2.3523 (6) Å, *ca* 0.15 Å longer than the corresponding Ni—Cl distance in [Tp^Ph2^NiCl], and consistent with the difference in the bromine and chlorine covalent radii (0.15 Å; Shannon, 1976). Interestingly, the Ni—Br bond length in (I) is significantly longer than that observed for [Tp*NiBr] (2.291 (2) Å). A similar increase, albeit not so marked, is also found between [Tp^Ph2^NiCl] and [Tp*NiCl] (ΔNi-Cl = 0.03 Å) suggesting that the larger Tp^Ph2^ ligand may be responsible for the longer nickel-halide bond distances.

The crystal packing in the structure of (I) contains several C—H···π interactions between the phenyl rings of neighbouring Tp^Ph2^ ligands (see Figure 2). The hydrogen atoms H6 and H11 are directed at the π bonds between C1—N1 and C4—C5, respectively {(C1—N1)π···H6 2.657 (3) Å; (C4—C5)π···H11 2.834 (5) Å} while H5 interacts with a phenyl ring (*Cg*1···H5 2.730 (3) Å; *Cg*1 is the centroid of ring C10—C15). Similar interactions occur on all three faces of the Tp^Ph2^ ligand creating a network of triangular columns such that all the [Tp^Ph2^NiBr] molecules point in the same direction and the phenyl rings adopt a propeller configuration.

### Experimental

NiBr_2_.2H_2_O (34 mg, 0.14 mmol) was dissolved in THF (5 ml) giving a green solution and then stirred for 5 min. KTp^Ph2^ (95 mg, 0.15 mmol) was dissolved in THF (5 ml) giving a pale yellow solution. Addition of the KTp^Ph2^ solution to the Ni solution resulted in a colour change to a red-pink solution. The solution was stirred for 16 hrs. The solution was reduced to dryness, redissolved in fresh THF (2 ml) and filtered through celite. The purple-pink solution was layered with hexanes (10 ml). After two days purple-pink blocks of (I) appeared. These were washed with EtOH (3 *x* 3 ml) and hexanes (2 *x* 5 ml) (59 mg, 54%). ν_max_(KBr)/cm^-1^ 3059w, 2966w, 2854w (νCH), 2626w (νBH). δ_H_ (300 MHz; CDCl_3_; SiMe_4_), 7.63 (br m, *m*- and *p*-Ph, 18H), 8.53 (br s, *o*-Ph, 6H), 8.86 (br s, *o*-Ph, 6H). UV–Vis λ_max_(CH_2_Cl_2_)/nm 318 (ε/dm^3^mol^-1^cm^-1^ 3140), 502 (600), 828 (120), 926 (160). m/*z* (ESI) 727 [M—Br^-^]^+^. Anal. Calc. for C_45_H_34_N_6_BBrNi [Tp^Ph2^NiBr]: C, 66.87; H, 4.24; N 10.40 Found: C, 66.62; H, 4.29; N, 10.17%

### Crystal data

**Table:**

[NiBr(C_45_H_34_BN_6_)]	*D*_x_ = 1.463 Mg m^−^^3^
*M**_r_* = 808.21	Mo *K*α radiation, λ = 0.71073 Å
Trigonal, *R*3	Cell parameters from 2851 reflections
Hall symbol: R 3	θ = 2.8–30.6°
*a* = 12.8227 (8) Å	µ = 1.66 mm^−^^1^
*c* = 19.327 (3) Å	*T* = 150 K
*V* = 2752.0 (5) Å^3^	Block, purple–pink
*Z* = 3	0.24 × 0.24 × 0.21 mm
*F*(000) = 1242	

### Data collection

**Table:**

Bruker SMART CCD diffractometer	2075 independent reflections
Radiation source: fine-focus sealed tube	1943 reflections with *I* > 2σ(*I*)
graphite	*R*_int_ = 0.037
Detector resolution: 100 pixels mm^-1^	θ_max_ = 27.5°, θ_min_ = 2.1°
φ scans	*h* = −16→16
Absorption correction: multi-scan (*SADABS*; Bruker, 1997)	*k* = −11→16
*T*_min_ = 0.691, *T*_max_ = 0.722	*l* = −21→25
5609 measured reflections	

### Refinement

**Table:**

Refinement on *F*^2^	Secondary atom site location: difference Fourier map
Least-squares matrix: full	Hydrogen site location: inferred from neighbouring sites
*R*[*F*^2^ > 2σ(*F*^2^)] = 0.028	H-atom parameters constrained
*wR*(*F*^2^) = 0.063	*w* = 1/[σ^2^(*F*_o_^2^) + (0.0169*P*)^2^] where *P* = (*F*_o_^2^ + 2*F*_c_^2^)/3
*S* = 1.06	(Δ/σ)_max_ = 0.001
2075 reflections	Δρ_max_ = 0.27 e Å^−^^3^
163 parameters	Δρ_min_ = −0.29 e Å^−^^3^
1 restraint	Absolute structure: Flack (1983), 670 Friedel pairs
Primary atom site location: structure-invariant direct methods	Flack parameter: 0.020 (8)

### Special details

**Table:**

Geometry. All e.s.d.'s (except the e.s.d. in the dihedral angle between two l.s. planes) are estimated using the full covariance matrix. The cell e.s.d.'s are taken into account individually in the estimation of e.s.d.'s in distances, angles and torsion angles; correlations between e.s.d.'s in cell parameters are only used when they are defined by crystal symmetry. An approximate (isotropic) treatment of cell e.s.d.'s is used for estimating e.s.d.'s involving l.s. planes.
Refinement. Refinement of *F*^2^ against ALL reflections. The weighted *R*-factor *wR* and goodness of fit *S* are based on *F*^2^, conventional *R*-factors *R* are based on *F*, with *F* set to zero for negative *F*^2^. The threshold expression of *F*^2^ > σ(*F*^2^) is used only for calculating *R*-factors(gt) *etc*. and is not relevant to the choice of reflections for refinement. *R*-factors based on *F*^2^ are statistically about twice as large as those based on *F*, and *R*- factors based on ALL data will be even larger.

### Fractional atomic coordinates and isotropic or equivalent isotropic displacement parameters (Å^2^)

**Table:**

	*x*	*y*	*z*	*U*_iso_*/*U*_eq_	
Br1	0.6667	0.3333	0.732391 (16)	0.02249 (14)	
Ni1	0.6667	0.3333	0.61068 (2)	0.01335 (14)	
N1	0.75319 (18)	0.26622 (19)	0.55312 (10)	0.0127 (4)	
N2	0.75175 (19)	0.29001 (19)	0.48407 (10)	0.0128 (4)	
B1	0.6667	0.3333	0.4567 (3)	0.0139 (10)	
H1B	0.6667	0.3333	0.3983	0.017*	
C1	0.8082 (2)	0.2003 (2)	0.55910 (12)	0.0146 (5)	
C2	0.8419 (3)	0.1815 (3)	0.49355 (13)	0.0166 (6)	
H2	0.8812	0.1373	0.4829	0.020*	
C3	0.8064 (2)	0.2405 (2)	0.44716 (12)	0.0136 (5)	
C4	0.8224 (3)	0.2532 (3)	0.37198 (14)	0.0135 (5)	
C5	0.7922 (2)	0.1509 (2)	0.33065 (13)	0.0173 (6)	
H5	0.7601	0.0738	0.3515	0.021*	
C6	0.8094 (3)	0.1632 (3)	0.25990 (16)	0.0182 (7)	
H6	0.7890	0.0942	0.2325	0.022*	
C7	0.8557 (2)	0.2743 (3)	0.22844 (13)	0.0201 (6)	
H7	0.8668	0.2814	0.1797	0.024*	
C8	0.8860 (3)	0.3759 (3)	0.26839 (13)	0.0215 (6)	
H8	0.9179	0.4525	0.2469	0.026*	
C9	0.8698 (3)	0.3652 (3)	0.33951 (14)	0.0190 (6)	
H9	0.8913	0.4350	0.3665	0.023*	
C10	0.8239 (2)	0.1510 (3)	0.62468 (13)	0.0163 (6)	
C11	0.8460 (2)	0.2131 (3)	0.68680 (14)	0.0185 (6)	
H11	0.8564	0.2919	0.6872	0.022*	
C12	0.8530 (3)	0.1605 (3)	0.74866 (17)	0.0230 (7)	
H12	0.8650	0.2023	0.7912	0.028*	
C13	0.8422 (3)	0.0472 (3)	0.74807 (16)	0.0269 (7)	
H13	0.8468	0.0113	0.7901	0.032*	
C14	0.8246 (3)	−0.0137 (3)	0.68574 (15)	0.0234 (7)	
H14	0.8191	−0.0904	0.6849	0.028*	
C15	0.8150 (3)	0.0381 (3)	0.62476 (16)	0.0209 (6)	
H15	0.8022	−0.0041	0.5824	0.025*	

### Atomic displacement parameters (Å^2^)

**Table:**

	*U*^11^	*U*^22^	*U*^33^	*U*^12^	*U*^13^	*U*^23^
Br1	0.02665 (19)	0.02665 (19)	0.0142 (2)	0.01332 (9)	0.000	0.000
Ni1	0.0140 (2)	0.0140 (2)	0.0120 (3)	0.00702 (10)	0.000	0.000
N1	0.0107 (10)	0.0149 (11)	0.0121 (9)	0.0061 (9)	−0.0007 (8)	0.0007 (8)
N2	0.0137 (11)	0.0146 (11)	0.0096 (8)	0.0068 (9)	0.0007 (8)	0.0006 (8)
B1	0.0112 (15)	0.0112 (15)	0.019 (2)	0.0056 (8)	0.000	0.000
C1	0.0121 (13)	0.0105 (13)	0.0199 (12)	0.0047 (11)	−0.0016 (10)	0.0005 (10)
C2	0.0186 (14)	0.0165 (15)	0.0191 (16)	0.0122 (13)	−0.0028 (13)	0.0009 (12)
C3	0.0097 (13)	0.0094 (12)	0.0189 (12)	0.0026 (11)	−0.0001 (9)	0.0010 (9)
C4	0.0106 (13)	0.0148 (14)	0.0169 (12)	0.0077 (11)	0.0010 (10)	−0.0012 (11)
C5	0.0171 (15)	0.0124 (14)	0.0221 (13)	0.0071 (12)	0.0005 (11)	0.0031 (11)
C6	0.0191 (16)	0.0155 (15)	0.0234 (16)	0.0113 (13)	−0.0015 (13)	−0.0039 (13)
C7	0.0180 (15)	0.0237 (15)	0.0153 (12)	0.0080 (13)	0.0044 (11)	0.0003 (11)
C8	0.0210 (15)	0.0179 (15)	0.0210 (12)	0.0062 (12)	0.0060 (11)	0.0053 (11)
C9	0.0199 (16)	0.0172 (15)	0.0193 (13)	0.0087 (13)	0.0016 (11)	−0.0029 (11)
C10	0.0089 (13)	0.0166 (14)	0.0215 (13)	0.0049 (11)	0.0001 (10)	0.0043 (11)
C11	0.0136 (14)	0.0155 (14)	0.0233 (13)	0.0050 (12)	−0.0050 (11)	0.0012 (11)
C12	0.0204 (17)	0.0291 (19)	0.0180 (15)	0.0112 (15)	−0.0034 (13)	0.0002 (14)
C13	0.0206 (16)	0.0304 (17)	0.0268 (14)	0.0105 (14)	−0.0022 (13)	0.0126 (13)
C14	0.0206 (16)	0.0195 (16)	0.0333 (16)	0.0124 (13)	−0.0008 (12)	0.0075 (13)
C15	0.0185 (15)	0.0194 (16)	0.0257 (14)	0.0101 (14)	−0.0019 (12)	−0.0011 (12)

### Geometric parameters (Å, °)

**Table:**

Ni1—Br1	2.3523 (6)	C5—H5	0.9500
Ni1—N1	2.041 (2)	C6—C7	1.380 (4)
Ni1—N1^i^	2.041 (2)	C6—H6	0.9500
Ni1—N1^ii^	2.041 (2)	C7—C8	1.392 (4)
N1—C1	1.350 (3)	C7—H7	0.9500
N1—N2	1.371 (3)	C8—C9	1.387 (4)
N2—C3	1.360 (3)	C8—H8	0.9500
N2—B1	1.544 (3)	C9—H9	0.9500
B1—N2^i^	1.544 (3)	C10—C11	1.389 (4)
B1—N2^ii^	1.544 (3)	C10—C15	1.394 (4)
B1—H1B	1.1278	C11—C12	1.397 (4)
C1—C2	1.398 (4)	C11—H11	0.9500
C1—C10	1.475 (3)	C12—C13	1.390 (5)
C2—C3	1.388 (3)	C12—H12	0.9500
C2—H2	0.9500	C13—C14	1.391 (5)
C3—C4	1.465 (4)	C13—H13	0.9500
C4—C9	1.397 (4)	C14—C15	1.388 (4)
C4—C5	1.415 (4)	C14—H14	0.9500
C5—C6	1.381 (4)	C15—H15	0.9500
			
N1—Ni1—N1^i^	93.11 (8)	C4—C5—H5	120.0
N1—Ni1—N1^ii^	93.11 (8)	C7—C6—C5	121.0 (3)
N1^i^—Ni1—N1^ii^	93.11 (8)	C7—C6—H6	119.5
N1—Ni1—Br1	123.04 (6)	C5—C6—H6	119.5
N1^i^—Ni1—Br1	123.04 (6)	C6—C7—C8	119.7 (2)
N1^ii^—Ni1—Br1	123.04 (6)	C6—C7—H7	120.1
C1—N1—N2	106.99 (19)	C8—C7—H7	120.1
C1—N1—Ni1	141.45 (17)	C9—C8—C7	119.9 (3)
N2—N1—Ni1	111.47 (15)	C9—C8—H8	120.0
C3—N2—N1	109.82 (19)	C7—C8—H8	120.0
C3—N2—B1	127.6 (2)	C8—C9—C4	120.9 (3)
N1—N2—B1	120.3 (2)	C8—C9—H9	119.5
N2—B1—N2^i^	108.89 (19)	C4—C9—H9	119.5
N2—B1—N2^ii^	108.89 (19)	C11—C10—C15	118.8 (3)
N2^i^—B1—N2^ii^	108.89 (19)	C11—C10—C1	121.9 (3)
N2—B1—H1B	110.0	C15—C10—C1	119.2 (2)
N2^i^—B1—H1B	110.0	C10—C11—C12	120.4 (3)
N2^ii^—B1—H1B	110.0	C10—C11—H11	119.8
N1—C1—C2	109.5 (2)	C12—C11—H11	119.8
N1—C1—C10	124.6 (2)	C13—C12—C11	120.1 (3)
C2—C1—C10	125.8 (2)	C13—C12—H12	120.0
C3—C2—C1	106.1 (2)	C11—C12—H12	120.0
C3—C2—H2	127.0	C14—C13—C12	119.8 (3)
C1—C2—H2	127.0	C14—C13—H13	120.1
N2—C3—C2	107.6 (2)	C12—C13—H13	120.1
N2—C3—C4	122.9 (2)	C15—C14—C13	119.7 (3)
C2—C3—C4	129.5 (2)	C15—C14—H14	120.2
C9—C4—C5	118.4 (2)	C13—C14—H14	120.2
C9—C4—C3	121.7 (3)	C14—C15—C10	121.1 (3)
C5—C4—C3	119.9 (2)	C14—C15—H15	119.4
C6—C5—C4	119.9 (3)	C10—C15—H15	119.4
C6—C5—H5	120.0		

Symmetry codes: (i) −*x*+*y*+1, −*x*+1, *z*; (ii) −*y*+1, *x*−*y*, *z*.

### Hydrogen-bond geometry (Å, °)

**Table:**

*D*—H···*A*	*D*—H	H···*A*	*D*···*A*	*D*—H···*A*
C5—H5···Cg1^iii^	0.95	2.73	3.589 (3)	151

Symmetry codes: (iii) −*x*+*y*+5/3, −*x*+1/3, *z*−2/3.
